# Carbapenemase-mediated resistance in *Acinetobacter baumannii* clinical isolates: molecular epidemiology and antimicrobial potential of chitosan nanoparticles

**DOI:** 10.1186/s12866-025-04706-w

**Published:** 2026-02-02

**Authors:** Fatma Abdullah, Ahmed F. Azmy, Zienab E. Eldin, Mai E. Ahmed, Fatma Molham

**Affiliations:** 1 Biotechnology and Life Sciences Department, Faculty of Postgraduate Studies for Advanced Sciences, Beni-Suef University, Beni-Suef, 62511 Egypt; 2 Microbiology and Immunology Department, Faculty of Pharmacy, Beni-Suef University, Beni-Suef, 62514 Egypt; 3 Materials Science and Nanotechnology Department, Faculty of Postgraduate Studies for Advanced Sciences, Beni-Suef University, Beni-Suef, 62511 Egypt; 4 Clinical Pathology Department, Faculty of Medicine, Beni-Suef University, Beni-Suef, Egypt

**Keywords:** Carbapenem resistance, Acinetobacter baumannii, Multidrug resistance, Chitosan nanoparticles, Antimicrobial activity

## Abstract

Antibiotic resistance is an escalating global health concern, and *Acinetobacter baumannii* remains one of the most challenging multidrug-resistant pathogens. This study investigated the prevalence of key carbapenemase genes among clinical *A. baumannii* isolates and evaluated the antimicrobial potential of chitosan nanoparticles (Cs NPs) as an alternative therapeutic approach. Thirty-five clinical isolates were screened for *blaOXA-51*, *blaOXA-23*, *blaNDM-1*, *blaIMP-1*, and *blaVIM-1* using colony PCR. Cs NPs were synthesized and characterized by XRD, FTIR, zeta potential, DLS, and TEM, and their antimicrobial activity was assessed using broth microdilution and electron microscopy. All isolates carried *blaOXA-51*, while *blaOXA-23* and *blaNDM-1* were detected in 80.0% and 45.7% of strains, respectively; *blaIMP-1* and *blaVIM-1* were absent. Cs NPs exhibited characteristic FTIR bands and an amorphous XRD profile, showed a narrow size distribution (~ 40 ± 2 nm), a zeta potential of + 35 ± 1 mV, and predominantly spherical morphology by TEM. They demonstrated strong antimicrobial activity, with MICs of 20–41.7 µg/mL and MBCs of 25–53.3 µg/mL, and TEM imaging revealed marked membrane disruption in treated cells. These findings highlight the high burden of carbapenemase genes in *A. baumannii* isolates and support Cs NPs as a promising antimicrobial candidate for combating this critical pathogen.

## Introduction

*Acinetobacter baumannii* represents a Gram-negative, non-fermentative cocco-bacillus that has emerged as a major causative agent of nosocomial infections [1]. The World Health Organization (WHO) ranks in the highest priority group of pathogens for the development of new antibiotics [2]. Moreover, it is listed as one of the six resistant “superbugs” of the “ESKAPE” pathogens by the Infectious Diseases Society of America [3]. This pathogen is capable of causing a wide variety of clinical manifestations like pneumonia, bacteremia, wound and burn infections, meningitis, and urinary tract infections [1]. The persistence of *A. baumannii* in healthcare settings is due to its capability to endure on surfaces by forming biofilm and its exceptional ability to acquire resistance to various medications [4].These features significantly contribute to its widespread prevalence and complicate eradication processes. Numerous factors contribute to the increasing rates of antibiotic resistance in *A. baumannii*, there is a direct correlation between the emergence of resistant strains and levels of antibiotic use [5]. Resistance mechanisms are transmitted from one bacterium to another, either longitudinally by inheritance from relatives or horizontally by plasmids; the latter can lead to transmission of resistance between different species [6].

Resistance mechanisms encompass reduced membrane permeability, increased efflux pump activity, target site modification via mutations or post-translational changes, and enzymatic inactivation of antibiotics through hydrolysis or chemical modification [1]. Among the principal contributors to multidrug resistance in *A. baumannii* are the β-lactamase enzymes, particularly oxacillinases and metallo-β-lactamases, which hydrolyze β-lactam antibiotics, including carbapenems, thus making them ineffectual [7]. The use of combination antibiotic therapy for infection treatment caused by MDR bacteria in an attempt to achieve synergistic benefits has occasionally resulted in the subsequent development of resistance mechanisms [8]. Therefore, Innovation in alternative therapeutic approaches is urgently needed to tackle this growing public health threat [9, 10].

Nanoparticles have gained significant attention as potential antimicrobial agents. Chitosan, which is derived from the deacetylation of chitin—one of the most abundant polysaccharides in nature—has a unique structure consisting of alternating 1–4 linked units of acetylglucosamine (2-acetamido-2-deoxy-β-d-glucopyranose) and glucosamine (2-amino-2-deoxy-glucopyranose) [11]. Antibacterial activity of chitosan nanoparticles (Cs NPs) is attributed to multiple mechanisms, including disruption of bacterial membrane integrity, penetration into bacterial cells, and intracellular interactions with vital biomolecules such as nucleic acid and proteins; these mechanisms act synergistically to impair bacterial viability and promote cell death [12]. In our study, we examined the prevalence of key carbapenemase genes *(blaOXA-23*, *blaNDM-1*, *blaVIM-1*, and *blaIMP-1*) among clinical *A. baumannii* isolates gathered in Egypt and evaluated the antimicrobial efficacy of chitosan nanoparticles (Cs NPs) against selected clinical strains.

## Materials and methods

### Sample collection and identification

Thirty-five non-duplicate clinical isolates of *A. baumannii* were obtained from the routine diagnostic microbiology laboratory of Beni-Suef University Hospital. Preliminary identification was performed using the Vitek 2 Compact system (BioMérieux, France). Genotypic confirmation was carried out by detecting the intrinsic *blaOXA-51* gene using colony PCR, and confirmed isolates were included for further analysis. Among these, we chose twelve isolates exhibiting resistance to more than eight antibiotics for subsequent experiments to assess the antibacterial activity of chitosan nanoparticles (Cs NPs).

### Antimicrobial sensitivity testing

 In vitro sensitivity assessments were determined using the disk diffusion method following the Clinical and Laboratory Standards Institute (CLSI) M100 [13]. Panel of 11 antimicrobial disc (HIMEDIA, India) were tested: Ampicillin/sulbactam (A/S,10/10 µg), piperacillin/tazobactam (PIT,100/10 µg), Cefepime(CPM,30 µg), Imipenem(IPM,10 µg), Gentamicin (GEN,10 µg), Amikacin (AK,30 µg), Doxycycline(DO,30 µg), Ciprofloxacin(CIP,5 µg), Levofloxacin(LE,5 µg), Trimethoprim/sulfamethoxazole(COT,25 µg), Ceftazidime(CAZ,30 µg).

### Detection of resistance genes by colony PCR

Colony PCR was performed as a rapid method to detect genes directly from bacterial colonies, without prior DNA extraction as described previously [14]. DNA templates were prepared by suspending 1–3 fresh colonies in 5 µL of sterile distilled water. PCR amplification was performed using specific primers of *blaOXA-51*, *blaOXA-23*, *blaNDM-1*, *blaIMP-1*, and *blaVIM-1* as shown in Table 1. A 10 µL of Mastermix (Ampliqon, Denmark), 0.5 µL of each reverse and forward primer (10 pmol), 5 µL of colony suspension, and 9 µL of sterile distilled water to complete the reaction volume (25 µL). With an initial denaturation at 95 °C for 15 min, 35 cycles of denaturation at 95 °C for 45 s, annealing for 45 s at primer-specific temperatures listed in Table 1, extension at 72 °C for 1 min, and a final extension at 72 °C for 10 min, the amplification reaction was conducted using a Biometra TProfessional 96 Thermocycler (Analytik Jena, Germany). Multiplex PCR was performed for *blaOXA-23* and *blaNDM-1*. PCR products were electrophoresed on 1.5% agarose gel containing ethidium bromide and finally visualized under UV Transilluminator (ECX-F20.M.,France).

**Table 1:** List of primers pairs, sequences, references, and product size

Gene	Primer sequence	Product size (bp)	Annealing (◦C)	Reference
*blaOXA − 51*	R: TAA TGC TTT GAT CGG CCT TG F: TGG ATT GCA CTT CAT CTT GG	353	50	[15]
*blaOXA-23*	R: ATGGAAGGGCGAGAAAAGGT F: ATCCATTGCCCAACCAGTCT	361	51	[16]
*blaIMP-1*	R: ATGTAAGTTTCAAGAGTGATGC F: ACGGTCTTGGCTATTTGGGG	568	46.5	[17]
*blaVIM-1*	R: GGTGTTTGGTCGCATATCGCAA F: ATTCAGCCAGATCGGCATCGG	502	58	[17]
*blaNDM-1*	R: CGGAATGGCTCATCACGATC F: GGTTTGGCGATCTGGTTTTC	521	51	[17]

*F* Forward primer, *R* Reverse primer

### Reagents and materials for chitosan nanoparticles (Cs NPs) preparation

Chitosan (Cs) with a molecular weight between 200 and 300 kDa and a high degree of deacetylation (> 90%) was purchased from Sigma-Aldrich (St. Louis, MO, USA).Naringenin (NRG), exhibiting a purity exceeding 98%, along with glacial acetic acid (99.7%), sodium tripolyphosphate (TPP, > 98%), and absolute ethanol (99.5%), were all obtained from Merck KGaA (Darmstadt, Germany) and were of analytical grade. All experimental procedures utilized deionized water.

### Preparation of chitosan nanoparticles (Cs NPs) by microfluidic-assisted ionic gelation

Chitosan nanoparticles were synthesized using a novel microfluidic-assisted ionic gelation method, which offers precise control over mixing conditions, leading to enhanced nanoparticle homogeneity and size distribution compared to traditional batch methods A 1 mg/mL chitosan solution was prepared by dissolving Cs in 100 mL of 0.6% (v/v) aqueous acetic acid, stirred at 1000 rpm overnight at room temperature homogeneous viscous solution. This solution was then filtered through a 0.45 μm syringe filter (Sartorius, Germany) to remove undissolved particulates and stored at 4 °C. Concurrently, a 1% (w/v) TPP solution was prepared in deionized water, used freshly prior to synthesis. For nanoparticle formation, both the chitosan and TPP solutions were loaded into separate 10 mL glass syringes and connected to the inlets of a custom-designed polydimethylsiloxane (PDMS) microfluidic chip featuring a Y-junction channel design (100 μm width × 50 μm height). Independent syringe pumps (Harvard Apparatus PHD 2000) were used to accurately control the flow rates, which were 50 µL/min for TPP and 100 µL/min for chitosan. The rapid and controlled mixing within the microfluidic channel facilitated instantaneous nanoparticle nucleation and growth, resulting in the formation of a cross-linked hydrogel network and the assembly of Cs NPs. Following microfluidic synthesis, the nanoparticle suspension was collected in a sterile tube and stirred at 800 rpm for 60 min to ensure complete formation and cross-linking. The nanoparticles were subsequently harvested using a Sigma 3-30KS centrifuge (Sigma Laborzentrifugen, Germany) at 15,000 rpm for 30 min at 4 °C. To remove residual TPP and unreacted components, the pellet was washed three times with deionized water. The resulting nanoparticles were subsequently lyophilized using a Labconco FreeZone 6 Freeze Dry System (Labconco, USA) at − 50 °C for 48 h, yielding a dry powder suitable for further analysis.

### Characterization of Cs nanoparticles (Cs NPs)

#### X-Ray diffraction (XRD)

The crystalline properties of Cs NPs were examined using a powder X-ray diffractometer (Bruker D8 Advance, Bruker Corporation, Germany). The instrument operated with Cu Kα radiation at a wavelength of 1.5406 Å, under conditions of 40 kV voltage and 40 mA current. Diffraction data were collected across a 2θ angular varies from 5° to 60°, by a scanning speed of 0.02° per second.

#### Fourier transform infrared spectroscopy (FTIR)

FTIR was carried out using a PerkinElmer Spectrum Two spectrometer (PerkinElmer, USA) to detect functional groups and confirm the chemical properties of Cs NPs. 200 mg of potassium bromide (KBr) and about 2 mg of the dried nanoparticle sample were mixed, crushed, and compressed into pellets. 32 scans of spectra were obtained for each sample, covering the 4000–400 cm⁻¹ range at a resolution of 4 cm⁻¹.

#### Particle size and zeta potential measurement

The particle size distribution, polydispersity index (PDI), and surface charge (zeta potential) of Cs NPs were determined using dynamic light scattering (DLS) and electrophoretic light scattering techniques on a Malvern Zetasizer Nano ZS (Malvern Instruments, UK). At a concentration of 1 mg/mL, lyophilized nanoparticles were reconstituted in deionized water and sonicated for five minutes before analysis. All experiments were carried out in triplicate to guarantee reproducibility, and measurements were made at 25 °C with a detection angle of 173°.

#### Transmission electron microscopy (TEM) analysis

Using a JEM-1400 transmission electron microscope (TEM) device (JEOL, Japan), the morphology of Cs NPs was investigated. This technique provided high-resolution images, enabling detailed assessment of nanoparticle size, shape, and surface characteristics at the nanoscale.

### Broth microdilution assay for MIC and MBC determination of chitosan nanoparticles

Minimum Inhibitory Concentration (MIC) was ascertained utilizing the broth micro-dilution technique within a 96-well microtiter plate according to the CLSI M07 guidelines [18]. Clinical isolates of *A.baumannii* were sub-cultured on Mueller–Hinton agar (MHA) (HIMEDIA, India) and incubated at 37 °C for 18–24 h. Bacterial suspensions were adjusted to 0.5 McFarland, subsequently diluted to a ratio of 1:20. Chitosan nanoparticles (Cs NPs) stock solution was prepared at 4 mg/ml and subjected to serial two-fold dilutions across the wells which contained 100 µL of Mueller–Hinton broth (MHB). They were then inoculated at 10% (v/v) with an inoculum that was around 10^6 CFU/mL and incubated for 18 h at 37 °C. Each MIC plate included positive growth control (inoculated MHB without Cs NPs) and negative sterility control (MHB only), and a Cs NPs (non-inoculated MHB with Cs NPs).

MIC was determined by determining the lowest concentration of chitosan nanoparticles that efficiently prevented visible bacterial growth. For determination of the Minimum Bactericidal Concentration (MBC), 10 µL aliquots from wells at MIC and higher were plated on agar plates and incubated for 24 h at 37 °C. After a 24-hour incubation period, the MBC concentration was calculated when no colonies were visible. All assays were conducted in triplicate.

### Growth Inhibition curve

According to CLSI M07 guidelines [18] Solutions, at the MIC and 0.5 MIC and 0.25MIC concentrations, were prepared and inoculated at 10% (v/v) using an inoculum of 10^^6^ CFU/mL and incubated at 37° C for 18 h in a microplate reader (Tecan sunrise, Austria). Optical density (OD₆₀₀) was recorded hourly. A positive control was drawn, for comparison purposes, using inoculated without chitosan nanoparticles. All assays were done in quadruplicate.

### Transmission electron microscope (TEM)

Bacterial samples were initially cultured on agar plates at 37 °C for 24 h under aerobic conditions. Subsequently, selected colonies were inoculated into tubes containing 3 mL of Luria-Bertani (LB) (HIMEDIA, India) broth and incubated at 37 °C for 3 h to reach the early logarithmic growth phase. Cs NPs were added to the bacterial suspension at a final concentration of 60 µg/mL. The mixture was then incubated for 2 h at 37 °C. Control samples were prepared without the addition of Cs NPs. After the incubation period, bacterial cells were harvested by centrifugation at 2000 rpm (approximately 200 × g) for 2 min. After carefully discarding the supernatant, the pelleted cells were fixed for two hours at room temperature using 15 µL of glutaraldehyde (2.5% v/v in phosphate buffer, pH 7.4). Following fixation, samples were sent to the Research Center for additional processing, such as critical point drying, dehydration, and electron microscopy analytical preparation. Following accepted procedures for bacterial electron microscopy, additional sample processing was carried out. In short, each sample was dehydrated by a series of graded ethanol (30%, 50%, 70%, 90%, and 100%) before being embedded in epoxy resin EPON 810 with DMP and polymerized for 48 h at 60 °C. After being cut into slices with an average thickness of 70 nm using a Leica ultramicrotome, the fixed samples were stained with uranyl acetate and lead citrate. After being put on copper grids, the samples were examined at 80 Kv using a JEOL JEM-1400 TEM (JEOLInc., Japan) [19].

### Statistical analysis

Data were analyzed using SPSS version [29.0.2.0] (IBM Corp., Armonk, NY, USA). Descriptive statistics were used to determine the prevalence of resistance genes. The Chi-square test (χ²) or Fisher’s exact test (when expected cell counts were < 5) was used to assess associations between resistance gene presence and phenotypic antibiotic resistance patterns. Mann-Whitney U test to compare the average Cs NPs MIC and MBC values between gene-positive and negative isolates. The MIC and MBC were expressed as mean from three independent experiments. *P*-values < 0.05 were considered statistically significant.

## Results

### Evaluation of antimicrobial sensitivity

**Fig. 1:**
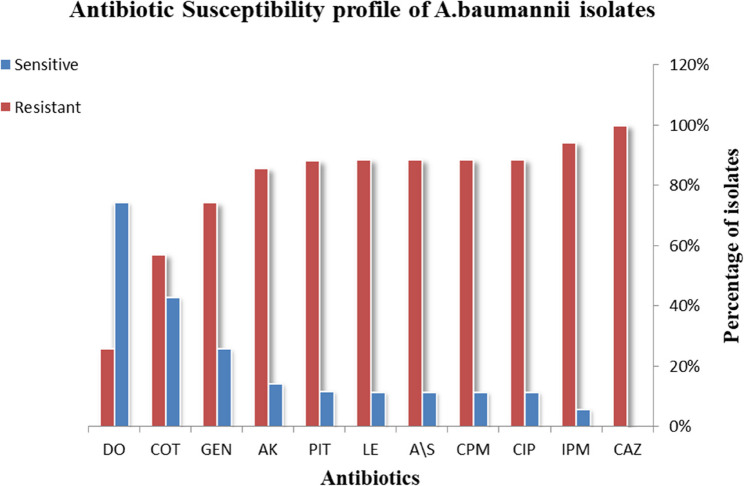
Antibiotics resistant patterns in *A. baumannii* isolates. (CPM) Cephepim; (CAZ) Ceftazidime; (DO**)** doxycycline; (AK) Amikacin; (GEN) Gentamicin; (CIP) Ciprofloxacin; (LE) levofloxacin; (PIT) Piperacillin-tazobactam; (A/S) Ampicillin- sulbactam; (COT) Trimethoprim-sulfamethoxazole; (IPM) Impenem

High levels of resistance to many antibiotics were observed in clinical isolates of *A.baumannii* when tested for antimicrobial susceptibility. Among the broad-spectrum cephalosporins, the isolates were highly resistant to Ceftazidime (100%) and Cefepime (89%). The isolates demonstrated great resistance to the carbapenem Imipenem (94%). Among aminoglycosides, high resistance rates were observed for Amikacin (86%) and Gentamicin (74%). Fluoroquinolones, including Ciprofloxacin (89%) and Levofloxacin (89%), also showed high resistance. For the β-lactam/β-lactamase inhibitor combinations, high resistance rates were observed with Ampicillin–sulbactam (89%) and Piperacillin–tazobactam (88%). Conversely, the isolates remained highly susceptible to the Doxycycline (74% susceptibility). Trimethoprim-sulfamethoxazole demonstrated moderate susceptibility (43%) as shown in Fig. 1.

### Prevalence of carbapenemase genes and their association with antibiotic resistance

**Fig. 2:**
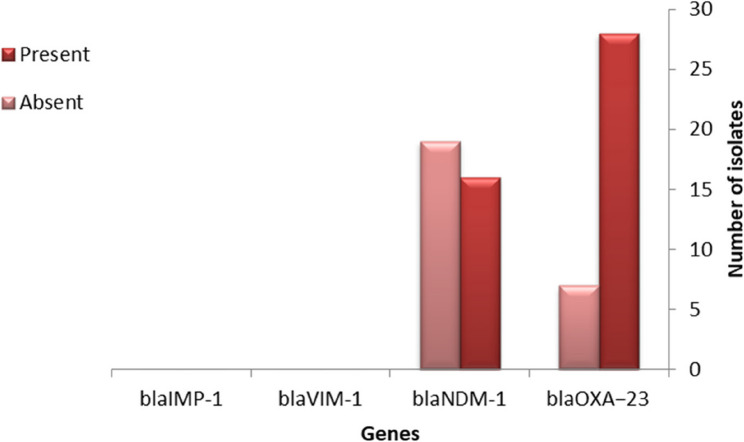
Prevalence of carbapenemase genes among 35 *A. baumannii*

**Fig. 3:**
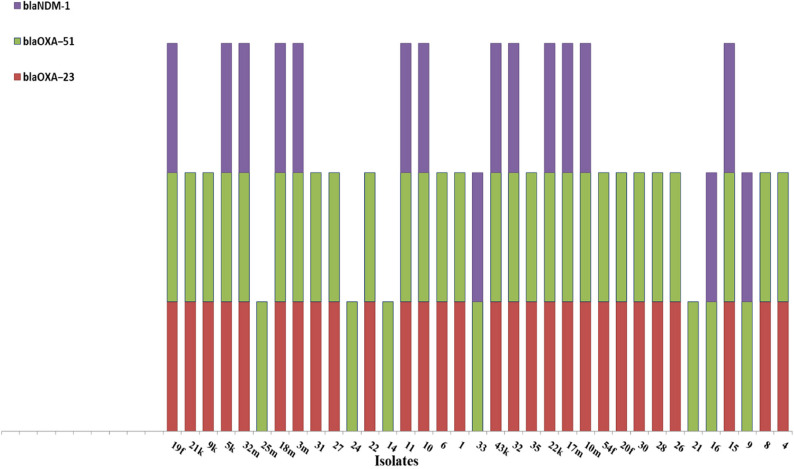
Co-occurrence of carbapenemase genes among 35 *A. baumannii* isolates

Thirty-five isolates were selected for PCR analysis targeting *blaOXA − 51*, *bla*OXA − 23, *blaNDM-1*, *blaIMP-1*, and *blaVIM-1* genes. Based on PCR results, all isolates (100%) carried the *blaOXA − 51* gene, the *blaOXA-23* gene was detected in 28/35 isolates (80.0%), and *blaNDM*-1 in 16/35 isolates (45.7%). Neither *blaIMP-1* nor *blaVIM*-1 was detected as shown in Fig. 2. Gene co-occurrence analysis revealed that 13 isolates (37.1%) harbored three carbapenemase genes Fig. 3. In order to evaluate potential correlations between the existence of resistance genes and phenotypic antimicrobial susceptibility, comprehensive statistical analyses were conducted. Significant associations were identified between *blaOXA-23* gene status and aminoglycoside resistance. Paradoxically *blaOXA-23* negative isolates demonstrated significantly higher resistance to Amikacin (AK) (χ² = 5.83, *p* = 0.016; Fisher’s exact *p* = 0.044). Additionally, a borderline correlation was observed between *blaOXA-23* and gentamicin (GEN) (χ² = 4.53, *p* = 0.033), although the results of the Fisher’s exact test suggested a value that was marginally non-significant (*p* = 0.055). No statistically significant correlations were found between *blaNDM-1* and resistance to any of the antibiotics tested.

### Characterization chitosan nanoparticles (Cs NPs)

**Fig. 4:**
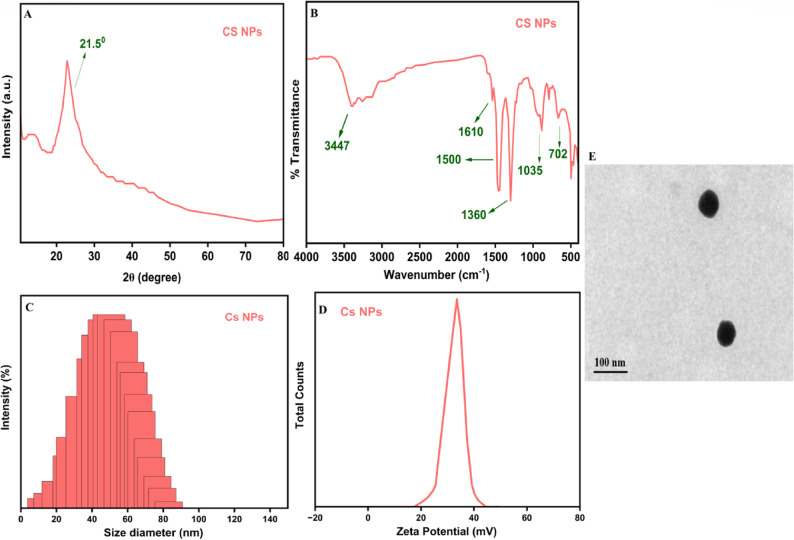
Physicochemical characterization of Cs NPs, (**A**) FTIR spectra, (**B**) XRD pattern, (**C**) Particle size analysis, (**D**) Zeta potential and (**E**) Transmission electron images of Cs NPs

FTIR spectroscopy (Fig. 4A) confirmed the successful formation of CS NPs, exhibiting characteristic peaks at 3447 cm⁻¹ (O-H and N-H stretching), 1610 cm⁻¹ (N-H bending of primary amine), 1500 cm⁻¹ (N-H bending of secondary amide), 1360 cm⁻¹ (C-H bending), 1035 cm⁻¹ (C-O stretching), and 702 cm⁻¹ (C-H bending). The X-ray Diffraction (XRD) pattern (Fig. 4B) displayed a broad peak at approximately 21.5°, which is characteristic of the amorphous nature of chitosan.

Dynamic Light Scattering (DLS) analysis, specifically the Zeta Sizer data (Fig. 4C), showed that the majority of the CS NPs had a size diameter 40 ± 2 nm, with a prominent peak around 30 nm, indicating a relatively narrow and desirable size distribution.

The Zeta Potential analysis (Fig. 4D) revealed a positive surface charge, with a single peak observed around + 35 ± 1 mV.

TEM analysis demonstrated that the synthesized Cs NPs exhibit a predominantly spherical morphology with smooth surfaces and an average diameter of approximately 70 nm (Fig. 4E).

### MIC and MBC

Cs NPs demonstrated potent antibacterial activity. MIC values against 12 clinical isolates ranged from 20 to 41.7 µg/mL, while MBC values ranged from 25 to 53.3 µg/mL as cleared in Table 2. Most isolates exhibited an MBC/MIC ratio ≤ 4, confirming bactericidal activity.

**Table 2:** Minimum inhibitory concentration (MIC) and minimum bactericidal concentration (MBC) of Chitosan nanoparticles (Cs NPs) against selected *A. baumannii* Isolates. The table shows the average of three independent experiments, MIC (µg /mL), MBC (µg /mL), and MBC/MIC ratios for the 12 highly resistant isolates

Isolate	Avg MIC	Avg MBC	MBC/MIC Ratio	Effect MBC/MIC ≤ 4
Isolate 10	30	41.7	1.39	Bactericidal
Isolate 22	30	41.7	1.39	-
Isolate 18 m	41.7	53.3	1.28	-
Isolate 25 m	30	53.3	1.78	-
Isolate 32 m	30	30	1	-
Isolate 19f	30	30	1	-
Isolate 31	20	41.7	2.08	-
Isolate 32	25	25	1	-
Isolate 5k	36.7	41.7	1.14	-
Isolate 21k	36.7	36.7	1	-
Isolate 17 m	41.7	41.7	1	-
Isolate 9k	41.7	41.7	1	-

### Growth inhibition assay

**Fig. 5:**
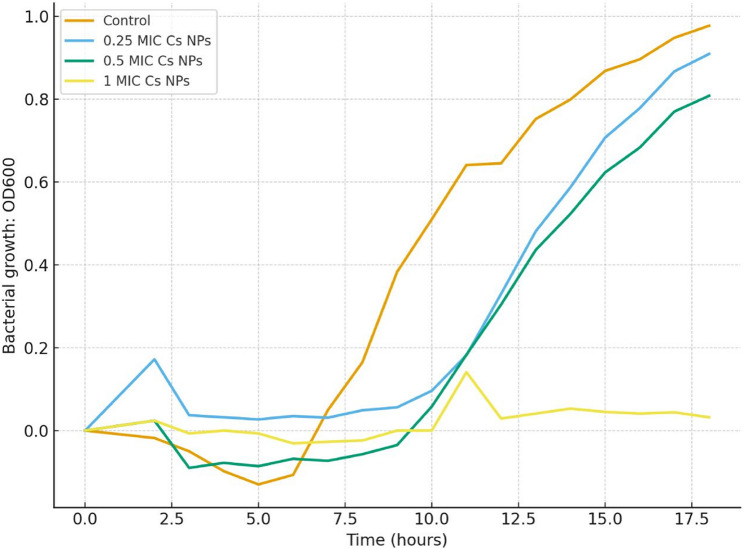
Growth Inhibition Curve of *A. baumannii* Isolates Exposed to Chitosan Nanoparticles (Cs NPs). The graph plots the Optical Density (OD) at 600 nm over 18 h of incubation at 37 °C. Control: Isolate growth without Cs NPs. Test Groups: Isolate growth in the presence of various Cs NP concentrations (.25MIC, .5MIC, and MIC)

Growth kinetics showed that Cs NPs clearly inhibited *A. baumannii* in a dose-dependent manner. Lower optical density measurements during the course of the 18-hour experiment showed that bacterial growth was inhibited by increasing chitosan concentrations. In particular, bacterial growth was successfully suppressed by a Cs NPs concentration of MIC. As shown in Fig. 5, bacterial growth was also considerably inhibited by lower Cs NPs concentrations of 0.5 MIC and 0.25 MIC in comparison to the control.

### TEM

**Fig. 6:**
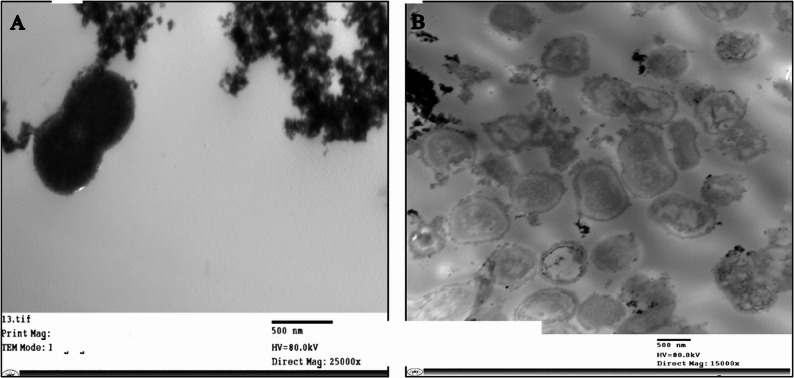
Transmission Electron Microscopy (TEM) Images Illustrating the Morphological Damage to *A. baumannii* by Chitosan Nanoparticles(Cs NPs). Untreated *A. baumannii* (**A**) vs. Cs NPs-treated bacteria **B**

TEM was used to evaluate the impact of Cs NPs on the intracellular contents and morphology of *A. baumannii* cultured without Cs NPs (negative control) showed definite cell walls and intact cell membranes. In addition to characteristic signs of active replication, such as the presence of septa (cross-wall formation), which indicate a normal and viable population, as shown in Fig. 6. In contrast, exposure to Cs NPs at 2× MIC for 120 min, ultrastructural alterations were observed, including shrinkage in cell membrane, cell lysis followed by cytoplasmic content leakage from the intracellular environment.

## Discussion

*Acinetobacter baumannii* exhibits a notable capacity to establish and maintain diverse mechanisms of antibiotic resistance, thereby positioning it as one of the most challenging nosocomial infections globally [5]. The current study added to our knowledge of resistance trends in the Middle East by exploring the molecular epidemiology of specific carbapenemase genes. Moreover, we assessed the activity of one of promising natural antimicrobial agent chitosan nanoparticles (Cs NPs).

The high prevalence of *blaOXA-23* (80%) among our resistant strains supports previous research, highlighting this enzyme’s contribution to antibiotic resistance, particularly when it is overexpressed or when multiple resistance mechanisms are active simultaneously [20, 21]. In contrast, *blaVIM-1* and *blaIMP-1*were not found. This absence likely mirrors local epidemiological trends, as the distribution of metallo-β-lactamase genes varies greatly depending on geographic and healthcare contexts. In various domains, the resistance of A. baumannii to carbapenems is predominantly attributed to the presence of OXA-type carbapenemases, rather than being associated with Class B metallo-β-lactamases [22].

In our research, we found a significant presence of *blaOXA-23*, which is in line with global epidemiological data. This gene is recognized as the most prevalent acquired OXA-type carbapenemase in A. baumannii on a worldwide scale [23]. This carbapenemase is prevalent in A. baumannii resistance patterns across various continents, including countries in Europe, Asia, and Latin America. Surveillance data from Greece, Indonesia, and Brazil demonstrate that 96.9%, 85.18%, and 95.4% of carbapenem-resistant isolates, respectively, carry *blaOXA-23* [23–25].

The detection of *blaNDM-1* in 45.7% of isolates is very alarming, as this gene has been increasingly implicated in outbreaks across the Middle East and Asia [26, 27]. Furthermore, the co-occurrence of *blaOXA-23* and *blaNDM-1* in certain isolates underscores the potential for the emergence of highly resistant profiles that leave clinicians with exceedingly limited treatment alternatives.

While most epidemiological studies indicate lower *blaNDM-1* prevalence compared to *blaOXA-23* in *A. baumannii*, certain regions and outbreak scenarios show elevated rates. Regional studies from Libya, Tunisia, Morocco, and Cuba reported *blaNDM-1* frequencies of 22.2%, 2.8%, 21.7%, and 11%, respectively [28–31]. In Nepal, *blaVIM-1* and *blaIMP-1* genes were detected in 17.7% and 16.8% of isolates [32], while Iraqi studies documented combined (*blaVIM-1* + *blaVIM-2*) and (*blaIMP-1* + *blaIMP-2*) prevalence rates of 57.1% and 34.1%, respectively [33].

Egyptian surveillance studies reveal variable resistance gene distributions. Hassan et al. [34] reported *bla-OXA-23*, *blaNDM-1*, and *blaVIM* prevalence rates of 77.7%, 11.7%, and 0.5%, respectively. Conversely, Al-Agamy et al. [35] found 50% *blaOXA-23* prevalence among carbapenem-insensitive strains from two Egyptian hospitals, with complete absence of MBL-encoding genes. A study conducted at Zagazig University reported that 90% and 66.7% of carbapenem-resistant A. baumannii (CRAB) isolates possessed *blaOXA-23* and *blaNDM* genes, respectively [36]. Additional investigations from Saudi Arabia and Egypt demonstrated universal *blaOXA-23* presence in all CRAB isolates [37, 38], with Kamel et al. reporting varying *blaNDM* and *blaVIM* rates of 12.1% and 100%, respectively.

Earlier Egyptian studies showed more modest *blaOXA-23* dominance, with AlHassan et al. [39] reporting 55.8% prevalence. Recent work by Koriem et al. [40] identified *blaOXA-23* in 94% of isolates while detecting no *blaNDM-1*genes in CRAB strains. These findings indicate substantial *blaOXA-23* and *blaNDM-1* prevalence across Egyptian healthcare facilities, particularly in tertiary care centers. This evidence supports the conclusion that class D β-lactamases constitute the primary carbapenemase type, with metallo-β-lactamases representing the secondary resistance mechanism. The concurrent detection of both *blaOXA-23* and *blaNDM-1* in 37.14% of our isolates signifies a concerning trend in epidemiological developments. This increasingly documented global phenomenon indicates the accumulation of resistance factors within transferable genetic elements, enabling concurrent dissemination and persistence. The combined action of multiple carbapenemases can generate exceptionally elevated resistance levels that challenge even recently developed antimicrobial compounds.

The microfluidic-assisted ionic gelation method employed for their preparation ensures the formation of stable nanoparticles with these desirable properties, which are crucial for optimizing their antimicrobial efficacy against a broad spectrum of microorganisms, including bacteria, fungi, and some viruse [41].The characterization of Cs NPs prepared by microfluidic-assisted ionic gelation revealed key insights into their structural, chemical, and physical properties. The FTIR analysis verified the successful formation of chitosan nanoparticles through ionic gelation. The characteristic amine and hydroxyl peaks confirmed involvement in cross-linking reactions with the polyanion (usually sodium tripolyphosphate, TPP). This demonstrates that the gelation mechanism was primarily mediated by electrostatic interactions, a hallmark of ionic gelation processes [42]. The broad XRD halo at 21.5° aligns with earlier reports on amorphous chitosan, suggesting that the microfluidic-assisted ionic gelation method prevents extensive crystallization. Maintaining amorphousness is beneficial since crystalline domains may limit molecular flexibility and reduce the availability of reactive functional groups critical for antimicrobial activity [42]. The narrow size distribution (30–40 nm, PDI < 0.2) is advantageous because nanoparticle uniformity ensures reproducibility in biological interactions. Smaller nanoparticles (< 50 nm) can readily penetrate microbial cell membranes, disrupt structural integrity, and in some cases enter intracellular compartments [43]. A consistent nanoscale diameter enhances pharmacokinetic predictability in drug delivery applications as well. The positive zeta potential (+ 35 mV) not only signifies commendable colloidal stability (preventing aggregation due to electrostatic repulsion) but also supports the antimicrobial efficacy. The polycationic nature of chitosan facilitates interactions with negatively charged components of microbial membranes, including lipopolysaccharides present in gram-negative bacteria and teichoic acids found in gram-positive bacteria; this electrostatic adhesion destabilizes the cell envelope, leading to leakage of proteins, nucleic acids, and ions, ultimately causing microbial cell death [44, 45]. The spherical morphology and nanoscale size with high surface-area-to-volume ratios enhance the contact area between Cs NPs and microbial cells, improving antimicrobial interactions. The observed mild aggregation can be attributed to van der Waals forces and drying during TEM sample preparation. Despite this, uniform morphology suggests that the microfluidics-assisted ionic gelation approach yields highly controlled nanostructures suitable for biomedical and antimicrobial purposes [46]. These physicochemical features particularly nanoscale size, positive surface charge, and uniform morphology likely underpin the enhanced antimicrobial potency observed in our study.

Our synthesized Cs NPs exhibited remarkable antimicrobial activity against *A. baumannii* isolates (MIC: 20–41.7 µg/mL; MBC: 25–53.3 µg/mL), demonstrating bactericidal properties with significant clinical potential against drug-resistant strains. When compared to previous studies, our results show markedly superior performance. Previous chitosan studies reported substantially higher MIC values (162–310 µg/mL) against *A. baumannii* [47], indicating our formulation achieved 4–15 fold greater potency. In another studies, the reported MIC values of 125 µg/mL [48] and 10–300 µg/mL [49], along with high MBC values of 500 µg/mL. The importance of nanoparticle formulation is emphasized by findings showing that while CS@Fe3O4 nanoparticles effectively reduced *A. baumannii* colony counts in both drug-sensitive and drug-resistant strains, chitosan alone at equal concentrations revealed no antibacterial activity [50].

The growth inhibition curves revealed concentration-dependent patterns, while sub-MIC levels showed partial inhibitory effects; the MIC concentration achieved complete inhibition. Treatments with Sub-MIC extended the lag phase which reduces the maximum growth rate. The observed growth patterns are in line with chitosan’s proposed mechanism of action, involving disruption of cell membrane integrity and interference with cellular functions and metabolic process. The growth recovery at 0.25 MIC after 8 h may indicate adaptive responses or degradation of Cs NPs over time [51, 52].

Transmission electron microscopy analysis supports our previous experimental findings. TEM imaging demonstrated progressive membrane disruption as the primary mode of chitosan action. Control bacteria exhibited intact cell walls with uniform membrane structure, while treated cells showed irregular membrane, cytoplasmic leakage, and there is complete cellular lysis. The observed damage supports chitosan’s proposed mechanism involving electrostatic interaction between its cationic amino groups and negatively charged bacterial cell surfaces [12]. These ultrastructural changes validate the growth inhibition patterns and physicochemical features of CS NPs, providing visual confirmation of their membrane targeting antimicrobial mechanism.

Notably, there is no statistically significant difference in the average MIC and MBC values between the presence and absence of *bla*Oxa-23 and *bla*NDM-1. This finding suggests that Cs NPs may serve as a promising adjunctive treatment for carbapenem-resistant A. baumannii, particularly in scenarios where the synthesis of β-lactamases limits the efficacy of conventional therapies.

Several limitations should be acknowledged, our focus on specific carbapenemase genes may have underestimated the contribution of other resistance mechanisms, including porin modifications, efflux pumps, and additional β-lactamases. Furthermore, the complex host environment, where Cs NPs function may be influenced by a variety of parameters like tissue penetration, immunological interactions, and protein binding, may not be adequately reflected by in vitro condition. Future studies should focus on pharmacokinetic analysis, toxicity assessments, and in vivo efficacy studies. Crucial data about the long-term durability of treatment could be gathered by employing serial passage testing to look into potential resistance development to Cs NPs.

## Conclusion

Our findings illuminate the multifaceted molecular epidemiology underlying carbapenemase-driven resistance patterns in *A. baumannii* strains from Egypt. microfluidic-assisted ionic gelation yielded stable, predominantly amorphous chitosan nanoparticles with a narrow nanoscale distribution, strong positive surface charge, and spherical morphology. These combined physicochemical features favor robust electrostatic interactions with microbial membranes, supporting effective broad-spectrum antimicrobial activity. The established antimicrobial efficacy of chitosan nanoparticles (Cs NPs) against these resistant isolates, irrespective of their resistance gene profiles, indicates a potentially viable strategy for addressing carbapenemase-mediated resistance. The multi-target mechanism of action, confirmed through ultra-structural analysis, provides hope for sustained efficacy against bacterial resistance mechanisms. These findings contribute to the rising evidence supporting nanotechnology-based approaches as promising solutions to the global antimicrobial resistance crisis.

## Data Availability

All data generated or analyzed during this study are included within this published article.
